# TOR‐mediated regulation of metabolism in aging

**DOI:** 10.1111/acel.12689

**Published:** 2017-10-02

**Authors:** Henri Antikainen, Monica Driscoll, Gal Haspel, Radek Dobrowolski

**Affiliations:** ^1^ Federated Department of Biological Sciences New Jersey Institute of Technology Rutgers University Newark NJ 07102 USA; ^2^ Department of Molecular Biology and Biochemistry Rutgers University Piscataway NJ 08854 USA

**Keywords:** aging, autophagy, lysosomal clearance, metabolism, mTOR

## Abstract

Cellular metabolism is regulated by the mTOR kinase, a key component of the molecular nutrient sensor pathway that plays a central role in cellular survival and aging. The mTOR pathway promotes protein and lipid synthesis and inhibits autophagy, a process known for its contribution to longevity in several model organisms. The nutrient‐sensing pathway is regulated at the lysosomal membrane by a number of proteins for which deficiency triggers widespread aging phenotypes in tested animal models. In response to environmental cues, this recently discovered lysosomal nutrient‐sensing complex regulates autophagy transcriptionally through conserved factors, such as the transcription factors TFEB and FOXO, associated with lifespan extension. This key metabolic pathway strongly depends on nucleocytoplasmic compartmentalization, a cellular phenomenon gradually lost during aging. In this review, we discuss the current progress in understanding the contribution of mTOR‐regulating factors to autophagy and longevity. Furthermore, we review research on the regulation of metabolism conducted in multiple aging models, including *Caenorhabditis elegans*,* Drosophila* and mouse, and human iPSCs. We suggest that conserved molecular pathways have the strongest potential for the development of new avenues for treatment of age‐related diseases.

## Introduction

The most studied and best understood longevity pathways govern metabolism according to available nutrient levels. The fundamental mechanisms from signaling cascades to protein complexes are conserved across phyla. A controlling hub at the center of nutrient sensing and signaling is the mechanistic target of rapamycin (mTOR) that governs cellular growth, protein synthesis, and degradation. mTOR acts upstream of several transcription factors, such as TFEB, FOXO, FOXA, and Nrf, that are essential for lifespan‐extending strategies such as dietary restriction. These transcription factors also control autophagy, a cellular process that clears proteins and dysfunctional organelles, and reduces proteotoxic and oxidative stress while maintaining a pool of amino acids for protein synthesis. mTOR responds to amino acids, a pathway modulated by proteins such as sestrins. Here we will review the current knowledge on the best‐known longevity pathways across animal models, namely insulin/insulin‐like signaling and its downstream transcription factor FOXO, and transcription factor FOXA‐dependent signaling. We consider how FOXO and FOXA are regulated by mTOR, and what role autophagy plays in the lifespan extension they confer. We also consider additional longevity mechanisms that rely on lipid signaling and the proteasome. We conclude with a discussion of how advancements in technologies such as induced pluripotent stem cells can enable the study of longevity‐regulating mechanisms in human systems, and how emerging ideas on nuclear‐cytoplasmic compartmentalization and its loss could contribute to our understanding of transcriptional dysregulation of nutrient‐sensing pathways in aging.

## mTOR kinase complexes and their regulation

mTOR is a member of the phosphoinositide 3‐kinase (PI3K)‐related protein family that constitutes two structurally and functionally distinct complexes, the mTOR complex 1 (mTORC1) and the mTOR complex 2 (mTORC2). The composition of both mTOR complexes is well characterized and has been reviewed elsewhere (Laplante & Sabatini, 2012). Specific inhibition of either mTOR complex requires the use of non‐pharmacological strategies such as RNAi‐mediated knockdown or genetic knockout of either their components Raptor (for mTORC1) or Rictor (for mTORC2). So far, no specific allosteric inhibitors are known and both complexes are susceptible to mTOR inhibitors such as Rapamycin or Torin (Hara *et al*., 2002; Sarbassov *et al*., 2006). Functionally, mTORC1 has been shown to predominantly tether to lysosomal membranes where it senses the levels of amino acids and growth factors to regulate cellular metabolic processes including protein and lipid synthesis, lysosomal biogenesis, and autophagy (Cafferkey *et al*., 1993; Kunz *et al*., 1993; Wullschleger *et al*., 2006; Laplante & Sabatini, 2012; Nnah *et al*., 2015). Whether mTORC1 can be activated or localized elsewhere, possibly by differing sets of environmental cues, remains to be determined. In contrast, mTORC2 has been demonstrated to be involved in cell survival, apoptosis, and proliferation (Dos *et al*., 2004; Sarbassov *et al*., 2005; Guertin *et al*. 2006; Jacinto *et al*., 2006; García‐Martínez & Alessi, 2008). The functions of both complexes have been associated with aging in multiple model systems (Table 1). Their regulation is reviewed in the following section.

**Figure 1:**
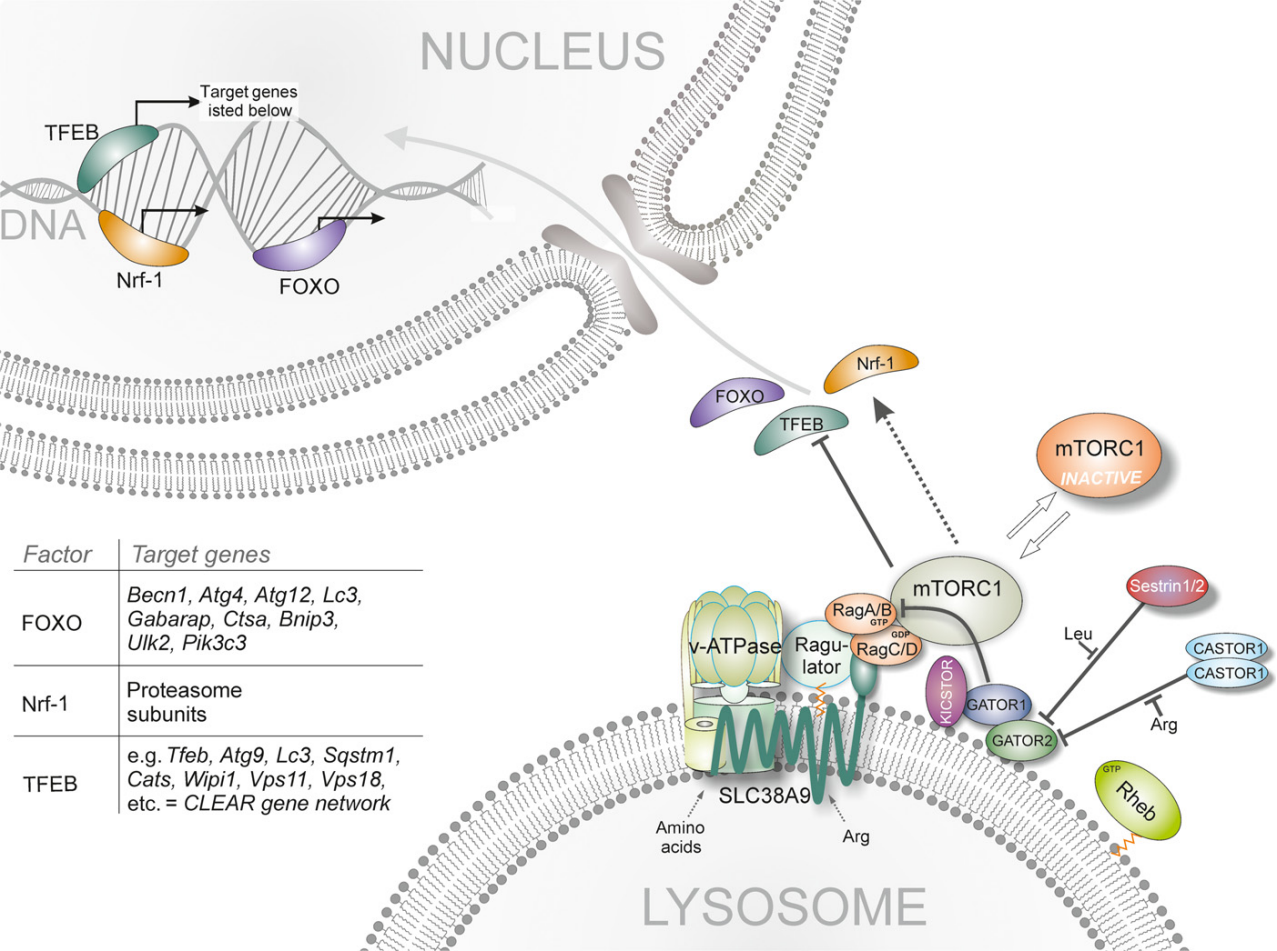
Transcriptional Regulation of Autophagy, Lysosomal, and Proteasomal Biogenesis through Lysosomal mTORC1. The so‐called lysosomal nutrient‐sensing (LYNUS) complex composed of vATPase, Ragulator, and the Rags (A/B and C/D) tether mTORC1 proximal to Rheb which activates the kinase complex. Membrane‐tethering of mTORC1 is regulated by the KICSTOR‐bounded GATOR complexes and the leucine‐sensing Sestrin 1/2 and arginine‐sensing CASTOR1 proteins. Active mTORC1 directly phosphorylates and inhibits the function of TFEB and FOXO while promoting Nrf‐1 activity associated with expression of proteasomal subunit. During starvation, mTORC1 dissociates from the lysosome and is rendered inactive. Under these conditions, TFEB and FOXO enter the nucleus to bind to their target promoters to promote lysosomal and autophagosomal gene expression, thereby restoring lysosomal activity through molecular clearance.

**Figure 2:**
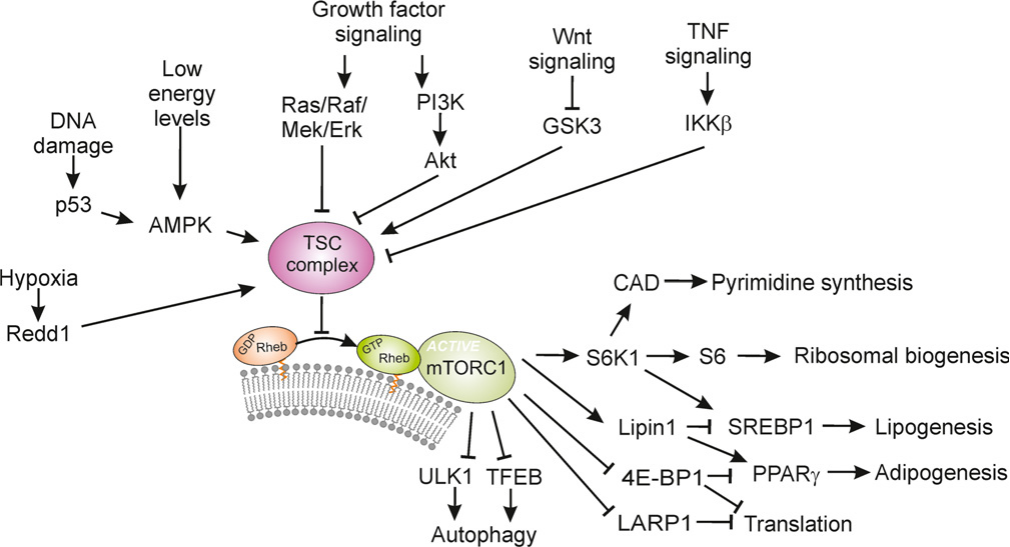
Regulation of Cellular Metabolism by Receptor‐Mediated Signaling, Tuberous Sclerosis (TSC) Complex, and mTORC1. The TSC complex is substrate to kinases and/or mediators regulated by incoming cellular signals ranging from hypoxia, DNA damage, and low energy levels to growth factors, Wnt, and TNF signals. Once phosphorylated, TSC complex dissociates from the lysosomal membrane to be degraded (Potter *et al*., 2002; Inoki *et al*., 2006; Demetriades *et al*., 2014; Menon *et al*., 2014), resulting in activating of Rheb. Active mTORC1 promotes pyrimidine synthesis (Yang *et al*., 2013a), ribosomal biogenesis (Thoreen *et al*., 2012), lipogenesis (Porstmann *et al*., 2008; Peterson *et al*., 2011), adipogenesis (Zhang *et al*., 2009), protein translation (Fonseca *et al*., 2015), and inhibition of lysosomal biogenesis (Sardiello *et al*., 2009) and autophagy (Kim *et al*., 2011; Settembre *et al*., 2011; Nazio *et al*., 2013).

While regulatory mechanisms of mTORC2 are currently debated and partially conflicting studies exist (Nobukuni *et al*., 2005; Frias *et al*., 2006; Jacinto *et al*., 2006; Tato *et al*., 2011; Yuan & Guan, 2015), mTORC2 has been associated with growth factor signaling and has been shown to be a downstream effector of PI3K signaling via the PH domain of its mSin1 subunit (Liu *et al*., 2015; Yuan & Guan, 2015). Active mTORC2 has also been shown to physically associate with ribosomes, suggesting that ribosomes activate mTORC2 directly and ensures that TORC2 is active only in growing cells (Zinzalla *et al*., 2011). Far more is known about the identity of protein complexes involved in the fine‐tuning of mTORC1 function. Major intracellular and extracellular signaling pathways including growth factor signaling, amino acids sensing, energy status monitoring, and cellular stress relays are transduced to the lysosome and integrate at mTORC1 (Fig. 1). Generally, mTORC1 activity is regulated by two successive events: translocation of the mTORC1 complex to the lysosomal surface, regulated by amino acid sensors, and mTORC1 activation by GTP‐bound Rheb, a state regulated by cellular signaling and stress (Fig. 2).

**Table 1:** TOR‐Signaling components or associated signaling molecules with demonstrated effect on lifespan

Gene	Relationship to TOR	Lifespan effect of inhibition	Process	Species	Reference
*let‐363*/*MTOR*	Complex component in mTORC1 and mTORC2	Up	TOR signaling	Yeast, *Caenorhabditis elegans*,* Drosophila*, Mouse	Vellai *et al*., 2003; Jia *et al*., 2004; Kaeberlein *et al*., 2005; Medvedik *et al*., 2007; Steffen *et al*., 2008; Tóth *et al*., 2008; Wei *et al*., 2008, 2009; Robida‐Stubbs *et al*., 2012
*rsks‐1*/*S6K1*	TOR substrate, promotes translation	Up	TOR signaling	Yeast, *Caenorhabditis elegans*,* Drosophila*, Mouse	Kapahi *et al*., 2004; Hansen *et al*., 2007; Pan *et al*. 2007; Sheaffer *et al*., 2008; Chen *et al*. 2009; Selman *et al*., 2009; Seo *et al*. 2013; McQuary *et al*. 2016
*TSC 1*/*2*	Upstream inhibitor of TOR	Down	TOR signaling	*Drosophila*	Kapahi *et al*., 2004; Zhang *et al*., 2017
*daf‐15*/*Raptor*	Adaptor for mTORC1	Up	TOR signaling	*Caenorhabditis elegans*	Jia *et al*., 2004; Chen *et al*. 2009; Seo *et al*. 2013
*skn‐1*/*Nrf*	Transcription factor, feedback modulator, downstream from TOR	Down	Proteasomal degradation	*Caenorhabditis elegans*	Robida‐Stubbs *et al*., 2012
*4E‐BP*	Translational repressor, downstream from TOR	Down	Protein synthesis	*Drosophila*	Zid *et al*. 2009
*eIF4F*	Initiation factor, downstream from TOR	Up	Protein synthesis	*Caenorhabditis elegans*	Pan *et al*. 2007
*daf‐16*/*Foxo*	Transcription factor, downstream from TOR	Down	IIS/TOR signaling	*Caenorhabditis elegans*	Lin *et al*., 1997; Lee *et al*., 2003; Murphy *et al*., 2003; Greer *et al*. 2007a; Chen *et al*. 2009; Honjoh *et al*., 2009; Robida‐Stubbs *et al*., 2012; Seo *et al*. 2013
*pha‐4*/*Foxa*	Transcription factor, downstream from TOR	Down	Dietary restriction	*Caenorhabditis elegans*	Panowski *et al*., 2007; Hansen *et al*., 2008; Sheaffer *et al*., 2008
*aak‐2*/*AMPK*	Converges with TOR on DAF‐16 (perhaps via TOR), inhibits mTORC1	Down	Energy metabolism	*Caenorhabditis elegans*	Greer *et al*. 2007a
*HLH‐30*/*TFEB*	Transcription factor, downstream from TOR	Down	Lysosomal biogenesis, autophagy	*Caenorhabditis elegans*	Lapierre *et al*., 2013
*daf‐2*/*INSR*	Converges with TOR on DAF‐16/FOXO	Up	IIS	*Caenorhabditis elegans*	Kimura *et al*. 1997; Tóth *et al*., 2008
*Raga‐1*	mTORC1 activator	Up	TOR signaling	*Caenorhabditis elegans*	Schreiber *et al*. 2010
*Rictor*	Component of mTORC2	Down	TOR signaling	*Caenorhabditis elegans*, Mouse	Soukas *et al*., 2009; Lamming *et al*., 2014

The mTORC1 complex is tethered to lysosomal membranes through heterodimeric RagA/B‐RagC/D GTPases in an amino acid‐dependent manner (Kim *et al*., 2008; Sancak *et al*., 2008). In particular, two amino acids, arginine and leucine, are necessary yet insufficient for mTORC1 activation (Bar‐Peled & Sabatini, 2014). Over the last few years, a number of proteins and protein complexes have been identified to convey information on amino acid availability to mTORC1. Among the first complexes described to have amino acid sensing function is the vATPase, a complex otherwise known as a vacuolar proton pump. The proposed mechanism describes an ‘inside‐out’ mechanism in which availability of lysosomal amino acids is relayed to the Ragulator complex, which binds to the mTORC1‐tethering Rag proteins (Sancak *et al*., 2008). Uptake of L‐leucine and other essential amino acids is mediated by a bidirectional transporter, the solute carrier 7A5/3A2 (SLC7A5/SLC3A2) that exchanges cellular L‐glutamine for extracellular L‐leucine (Nicklin *et al*., 2009). Several SLC proteins belonging to families capable of transporting amino acids at the plasma membrane have been shown to regulate mTORC1 activity (Nicklin *et al*., 2009). A recently characterized member of this family, the SLC38A9, is a lysosomal membrane protein interacting with the vATPase/Ragulator/Rag amino acid sensing complex required for its function (Rebsamen *et al*., 2015; Wang *et al*., 2015). In addition to vATPase and the SLCs, other protein complexes have been described to play significant roles in amino acid sensing. Leucyl‐tRNA synthetase (LeuRS), an enzyme known to promote loading of cognate tRNAs with leucine (Jakubowski, 2012), has been recently described by our group to form a constitutive complex with the tumor suppressor protein folliculin (FLCN) and GAP of RagC (Tsun *et al*., 2013; Khayati *et al*., 2017). Upon amino acid withdrawal, FLCN/LeuRS complex localizes to the lysosomal membrane, displacing mTORC1 into the cytoplasm. Interestingly, this complex is able to sense the levels of amino acid metabolite homocysteine, which accumulates in aging and constitutes a risk factor for sporadic Alzheimer's disease (Tucker *et al*., 2005). In *Caenorhabditis elegans,* LeuRS has recently been shown to be necessary for the lifespan increasing effect of a transcription factor network in response to germline removal, which effect is mediated by mTOR inhibition by LeuRS (Nakamura *et al*., 2016).

A group of mTOR‐regulating, stress‐sensing proteins associated with aging are sestrins. Sestrins are regulated by p53 and pCREB (sestrins 1 and 2) and the transcription factor FOXO (Sestrins 1 and 3) to inhibit mTORC1 through the so‐called GAP activity toward Rags (GATOR) complexes (Budanov & Karin, 2008; Budanov *et al*., 2010; Lee *et al*., 2010a; Lee *et al*., 2010b; Chantranupong *et al*., 2014; Reddy *et al*., 2016). Two GATOR complexes exist, the subcomplex GATOR1 harboring GAP activity toward the Rags, and GATOR2 which negatively regulates GATOR1 to render mTORC1 unresponsive to amino acids (Bar‐Peled *et al*., 2013). Cytosolic availability of leucine and arginine is directly sensed by sestrins (1 and 2) and cellular arginine sensor for mTORC1 (CASTOR) proteins, respectively (Wolfson *et al*., 2015; Chantranupong *et al*., 2016), to regulate GATOR/Rag complex activity and therefore mTORC1 tethering to lysosomal membranes (Fig. 1).

Once tethered, mTORC1 is activated by the membrane‐bounded Ras homolog enriched in brain (Rheb) protein (Inoki *et al*., 2003; Tee *et al*., 2003), which is in turn regulated by tuberous sclerosis‐1 and 2 (TSC1/2) complex, also known as hamartin/tuberin, a hub for several upstream signals that impact mTORC1 activity. These signals are conveyed by insulin‐like growth factor 1 (IGF1) receptors and its downstream effectors, such as PI3K and Ras pathways, protein kinase B (Akt/PKB), extracellular signal‐regulated kinase 1/2 (ERK1/2), and the ribosomal S6 kinase (S6K1) that directly phosphorylate and inactivate TSC1/2 (Manning *et al*., 2002; Roux *et al*., 2004). Another major pathway that activates mTORC1 through TSC1/2 is canonical Wnt signaling, which acts by inhibiting glycogen synthase kinase 3β (GSK3β) via an endolysosomal‐dependent process (Taelman *et al*., 2010; Dobrowolski & De Robertis, 2012) that phosphorylates and promotes TSC1/2 activity (Inoki *et al*., 2006). Furthermore, pro‐inflammatory cytokines such as tumor necrosis factor‐α (TNF‐α) modulate mTORC1 signaling (Lee *et al*., 2007) and consequently alter proliferation, differentiation, or apoptosis in a cell type‐specific manner. Cellular stress such as low energy, DNA damage, and hypoxia acts in part through TSC1/2 to inhibit mTORC1 activity either directly through adenosine monophosphate‐activated protein kinase (AMPK) (Inoki *et al*., 2003) or Redd1 (Brugarolas *et al*.,), respectively (Fig. 2).

Inhibiting mTOR signaling by pharmacological or genetic means has been shown to extend lifespan or healthspan in a host of species, from yeast (Kaeberlein *et al*., 2005; Powers *et al*., 2006), *Caenorhabditis elegans* (Vellai *et al*., 2003; Hars *et al*., 2007; Lapierre *et al*., 2011), and *Drosophila melanogaster* (Kapahi *et al*., 2004) to mice (Harrison *et al*., 2009; Wu *et al*., 2013) and dogs (Urfer *et al*., 2017). Some regimes of dietary restriction do not exhibit an additive increase on lifespan with mTOR inhibition, implying the underlying mechanisms for how they exert their influence on longevity are shared and likely driven by reduced TOR signaling (Kapahi *et al*., 2004; Kaeberlein *et al*., 2005; Hansen *et al*., 2007). However, pharmacological inhibition of mTOR in higher organisms, including humans, is associated with effects such as immunosuppression, the current medical use for rapamycin in organ transplant patients, and insulin insensitivity (Lamming *et al*., 2012). An in‐depth understanding of mTORC1‐specific nutrient‐sensing pathways could help developing more selective drugs that can mediate the preferred outputs from mTOR signaling, from preventing age‐related disease and promoting health.

## Conserved nutrient‐sensing pathways and their transcriptional effectors

It has been known that nutrient‐sensing pathways play a crucial role in longevity since the first lifespan‐extending mutations were discovered in *Caenorhabditis elegans*. The mutations in question, in the genes *age‐1* and *daf‐2*, targeted key components of insulin/insulin‐like growth factor‐1 signaling (IIS), namely PI3K in the case of the former and the homolog of insulin/insulin‐like growth factor receptor‐1 (INSR) in case of the latter. The discovery of lifespan extension by *daf‐2* mutations was followed by downstream analysis revealing that the effects of loss‐of‐function mutations in *daf‐2* require the activity of the gene *daf‐16* (Lin *et al*., 1997), a hepatocyte nuclear factor 3/forkhead transcription factor and the lone FOXO homolog in *Caenorhabditis elegans*, whose human homolog is regulated by insulin signaling. DAF‐16 causes metabolic changes similar to insulin and the expression of several longevity promoting genes, including heat shock proteins and antioxidants (Ogg *et al*., 1997; Lee *et al*., 2003; Murphy *et al*., 2003).

Since its discovery, the IIS pathway has been shown to regulate lifespan in other species, including *Drosophila* (Tatar *et al*., 2001; Hwangbo *et al*., 2004) and mice (Selman *et al*., 2008, 2011). Recently, the FOXO gene family has been identified as a candidate for human longevity (Lunetta *et al*., 2007; Willcox *et al*., 2008; Anselmi *et al*., 2009; Flachsbart *et al*., 2009; Li *et al*., 2009). Specifically, FOXO3A has a strong association with phenotypes including not only long lifespan, but also a long healthspan, as measured by readouts such as incidence of cardiovascular disease and cancer, self‐reported health and integrity of physical and cognitive functions (Willcox *et al*., 2008; Anselmi *et al*., 2009; Flachsbart *et al*., 2009). However, such findings may not hold in all human populations, as exceptions have been found (Kleindorp *et al*., 2011).

IIS is linked to mTOR signaling by several connections and downstream convergences. IIS activates Akt, which activates mTORC1 by reducing the activity of its inhibitor TSC2 (Inoki *et al*., 2002; Manning *et al*., 2002). Additionally, IIS activates mTORC2 via its mSin1 PH domain binding to PIP3 generated by plasma membrane PI3K; in turn, mTORC2 activates Akt (Liu *et al*., 2015; Yuan & Guan, 2015). mTORC1 negatively regulates IIS by two means: by activating the IIS inhibitor Grb10 (Hsu *et al*., 2011; Yu *et al*., 2011), and by promoting S6K1‐dependent degradation of insulin substrate 1, thus attenuating mTORC2 activity (Harrington *et al*., 2004; Shah *et al*., 2004).

IIS and mTOR signaling have also been shown to share two longevity‐regulating transcriptional effectors, namely SKN‐1/Nrf and DAF‐16/FOXO (Honjoh *et al*., 2009; Robida‐Stubbs *et al*., 2012; Ewald *et al*., 2015). Genetic inhibition of mTORC1 leads to an adaptive transcriptional response by SKN‐1/Nrf and DAF‐16/FOXO that extends lifespan in *Caenorhabditis elegans* (Robida‐Stubbs *et al*., 2012). While SKN‐1/Nrf signaling upregulates mTORC1 expression in a positive feedback loop, DAF‐16/FOXO has been shown to create a negative feedback loop for mTORC1 signaling by inhibiting DAF‐15/Raptor expression (Jia *et al*., 2004). Inhibiting mTOR signaling by rapamycin, which is less specific and also inhibits mTORC2 when administered chronically, induces lifespan extension dependent only on SKN‐1/Nrf (Robida‐Stubbs *et al*., 2012). Thus, the identity of the activated mTOR downstream transcription factors may depend on the signaling mTOR complex identity, with mTORC1 affecting both SKN‐1/Nrf and DAF‐16/FOXO activities, and mTORC2 being apparently more specific to SKN‐1/Nrf. In another study, mTORC2 has been shown to influence the activity of SKN‐1/Nrf via the serum‐ and glucocorticoid‐regulated kinase (Mizunuma *et al*., 2014). The mTOR complex specificity of downstream transcription factors could depend on environmental cues, as data contradicting the results by Robida‐Stubbs *et al*. (2012) have been obtained from developmental studies, where SKN‐1/Nrf has been shown to be regulated by mTORC2 but by not mTORC1 (Ruf *et al*., 2013), and mTORC2 to be essential to DAF‐16/FOXO signaling (Guertin *et al*., 2006). It is possible that the DAF‐16/FOXO activation in response to mTORC1 inhibition is mediated by IIS, as RHEB‐1 and TOR signaling mediate the downregulation of the insulin‐like peptide INS‐7 expression and IIS signaling in intermittent fasting (Honjoh *et al*., 2009). Another connection between TOR signaling and DAF‐16/FOXO is AAK‐2/AMPK, which is required for the synergistic lifespan extension by *daf‐2/INSR* and *rsks‐1/S6K1* double mutants (Chen *et al*., 2013), and could thus be a mediator of TOR signaling to DAF‐16/FOXO. Studies of transcriptional downstream effectors of mTOR and the connections of mTOR signaling to IIS would benefit from TOR complex‐specific inhibitors, as for example the difference obtained by genetic ablation and the use of rapamycin could also be due to some mTOR activities being seemingly rapamycin‐insensitive (Thoreen *et al*., 2009).

The discovery of the role of IIS in the modulation of longevity was the result of applying the most successful strategy to extend lifespan thus far, dietary restriction (Klass, 1977, 1983; Kenyon *et al*., 1993), and trying to understand its mechanisms. Dietary restriction has been shown to work as an anti‐aging intervention in several animal models, including primates, in which a recent re‐examination of two previous parallel studies in rhesus monkeys reconciled their seemingly contradictory data, and demonstrated the effectiveness of dietary restriction in extending lifespan and maintaining health (Mattison *et al*., 2017). As dietary restriction affects both IIS and mTOR signaling, it is of importance to delineate the contribution of each to overall lifespan extension. As mentioned, further reduction in TOR activity does not provide additional lifespan extension in some regimens of dietary restriction, as seen in yeast and worms (Kaeberlein *et al*., 2005; Hansen *et al*., 2007), or protect from lifespan reduction by dietary enrichment (Kapahi *et al*., 2004), suggesting mTOR‐mediation of the lifespan effect of some forms of dietary restriction in these species. However, in another study in flies, rapamycin was able to further increase the lifespan in mutants with weakened IIS, and also in animals under dietary restriction, suggesting differences in mechanisms of lifespan extension between TOR inhibition, IIS, and dietary restriction (Bjedov *et al*., 2010). Additionally, *rsks‐1/S6K1, aak‐2/AMPK,* and *daf‐2/INSR* mutations have been shown to have synergistic effects on lifespan (Chen *et al*., 2013; Hou *et al*., 2016). While these results suggest independent contributions to lifespan extension, definite conclusions cannot be made from double mutants involving loss‐of‐function mutations in *daf‐2/INSR*, as they are hypomorphic. A downstream distinction between IIS and TOR signaling is demonstrated by the fact that the lifespan extension provided by DAF‐2/INSR depletion requires DAF‐16 (Lin *et al*., 1997; Ogg *et al*., 1997), whereas increase to lifespan provided by TOR depletion does not (Vellai *et al*., 2003), showing that these two pathways can have distinct downstream mediators of lifespan effects in addition to the shared ones discussed above. Further studies of the activating cues of these pathways and their downstream targets are needed to elucidate the full degree of overlaps and distinctions between these longevity‐modulating pathways.

FOXA is a transcription factor that mediates lifespan‐extending effects of dietary restriction, and is independent of IIS, but connected to mTOR signaling. The *Caenorhabditis elegans* ortholog for FOXA, *pha‐4*, is required for the lifespan extension by dietary restriction (Panowski *et al*., 2007; Hansen *et al*., 2008), but not the extension caused by reduced IIS via *daf‐2/INSR* mutants, showing that PHA‐4/FOXA is part of a distinct pathway from IIS. Increases in lifespan by reduced TOR signaling require PHA‐4/FOXA (Sheaffer *et al*., 2008) and are mediated by the gene *rsks‐1*, encoding the homolog of the mammalian SK61, showing that FOXA is a necessary downstream component of TOR‐mediated lifespan increase. Lifespan extension by loss‐of‐function mutations targeting *rsks‐1/S6K1*, which is activated by mTOR, requires PHA‐4/FOXA (Sheaffer *et al*. 2008), providing further evidence that mTOR signaling exerts inhibition on PHA‐4/FOXA. Exactly how and through which intermediates this control occurs remains to be determined.

The energy level sensor AMPK regulates mTORC1 activity and shares downstream effectors of lifespan modulation with mTOR. AMPK responds to low levels of AMP and has been demonstrated to have a role in the lifespan extension in response to dietary restriction (Greer *et al*., 2007a), which is mediated at least in part by DAF‐16/FOXO. AMPK can also regulate mammalian FOXO3 (Greer *et al*., 2007b). In addition, overexpression of AMPK has been shown to extend lifespan in *Caenorhabditis elegans* (Greer *et al*., 2007a). Active AMPK downregulates mTORC1 activity indirectly by phosphorylating the serine sites on TSC2 (Inoki *et al*., 2003), and directly by phosphorylating Raptor (Gwinn *et al*., 2008). Additionally, the AMPK activating drug metformin, commonly prescribed for diabetic patients, has been shown to increase lifespan in *Caenorhabditis elegans* (Onken & Driscoll, 2010; Cabreiro *et al*., 2013) and mice (Martin‐Montalvo *et al*., 2013), but not in *Drosophila* (Slack *et al*., 2012). At least some of the lifespan‐extending effects of AMPK could be due to its inhibition of mTORC1, and metformin has been shown to act on mTOR signaling via Redd1 also independently of AMPK (Ben *et al*., 2011). AMPK modulation of lifespan has been shown to occur also via CREB‐regulated transcriptional coactivators (Mair *et al*., 2011). It is also notable that AMPK converges with mTOR signaling on ULK1 in the regulation of autophagy (Kim *et al*., 2011), which means autophagy‐mediated longevity extension could be promoted to an extent by either pathway, though of the two, only mTOR exerts transcriptional control over autophagy.

mTOR exerts energy‐ and nutrient‐sensitive control on mitochondrial gene expression and respiration by activating the peroxisome proliferator‐activated receptor coactivator‐1 α (PGC‐1 α) (Cunningham *et al*., 2007), which forms a complex with the transcription factor Yin‐Yang 1 (YY1) to promote related gene expression. mTOR‐mediated enhancement of mitochondrial respiration has been shown to increase lifespan in yeast (Bonawitz *et al*., 2007; Pan *et al*., 2011), in which part of the effect seems to be due to ROS‐mediated hormesis during glycolytic growth, and to increase leanness in adipose tissue‐specific raptor knockout mice (Polak *et al*., 2008). Additionally, mTOR can protect cells from defective mitochondria during aging by inducing autophagy, which can target mitochondria in a process called mitophagy (Palikaras *et al*., 2015). In addition to PGC‐1 α signaling and regulating mitophagy, mTOR can possibly also influence mitochondria via SKN‐1 signaling (Palikaras *et al*., 2015). While several studies have shown that mitophagy can extend lifespan (Schiavi *et al*., 2015; Ryu *et al*., 2016), the role of mTOR‐mediated mitophagy in longevity remains to be determined.

The final mTOR‐linked, nutrient‐sensing system included in this review is the eIF2α kinase general control nonderepressible 2 (GCN2), an amino acid sensing kinase. As GCN2 targets eIF2α, the major protein translation promoting kinase, GCN2 can affect protein synthesis rates (Berlanga *et al*., 1999). GCN2 has been shown to be required for extended longevity in response to amino acid deprivation (Kang *et al*., 2016), and to target the cap‐dependent translational inhibitor 4E‐BP, which is also controlled by mTOR. This raises the question as to whether mTOR could similarly mediate longevity in response to deprivation of amino acids to which it is sensitive. Interestingly, GCN2 and mTOR share another target in the form of activating transcription factor 4 (ATF4) (Park *et al*., 2017), which is involved in endoplasmic reticulum stress and the integrated stress response, and also mediates expression of 4E‐BP and amino acid transporters. Amino acid starvation induces eIF2α‐dependent ATF4 expression, whereas growth factor deprivation depletes ATF4. This model suggests that ATF4 upregulation attempts to provide amino acids for translation only if the growth factor/mTOR‐mediated demand for protein synthesis is present, by its transcriptional program that includes amino acid transporters and tRNA aminoacyltransferases as its targets. The complexity of this regulatory pathway is just starting to be understood, as ATF4 is also a component of the mTOR‐regulated, TFEB‐mediated integrated stress response (Martina *et al*., 2016), and ATF4 and TFEB target genes (for amino acid transporters and RagD, respectively) upregulate mTORC1 activity in a positive feedback loop.

## Amino acid and energy metabolism in longevity

There is evidence that the pathways mediating longevity in dietary restriction are engaged differently by different dietary restriction regimens (Greer *et al*., 2007a; Panowski *et al*., 2007; Greer & Brunet, 2009; Kenyon, 2010). It remains to be determined, especially in mammals, what the contribution of individual nutritional components is to the effects of dietary restriction, and what nutritional sensors and pathways are involved in mediating the effects. Recent work has implicated mTORC1 signaling in the effects of protein restriction in improved hepatic insulin sensitivity and stress resistance in mice (Lamming *et al*., 2015), and inhibition of cancerous growth in tumor xenografts (Roos *et al*., 2009). Interestingly, protein restriction is sufficient to reduce IGF‐1 signaling in humans (Fontana *et al*., 2008). In *Drosophila*, protein restriction has been shown to play a greater role in lifespan extension than simple caloric restriction (Mair *et al*., 2005), an effect mostly dependent on essential amino acids (Grandison *et al*., 2009). Based on lifespan studies in rodents (Zimmerman *et al*., 2003; Miller *et al*., 2005), and on what is known about the amino acid sensing pathways via mTOR for arginine and leucine (reviewed here) and GCN2 for tryptophan (Peng *et al*., 2012), it seems possible that individual amino acids may have crucial roles in controlling the activity of lifespan modulating signaling pathways, as shown also by the lifespan extension provided by methionine restriction (Miller *et al*., 2005).

In addition to amino acids, amino acid metabolites may modulate lifespan. We have recently shown that the methionine metabolite homocysteine can upregulate mTORC1 activity (Khayati *et al*., 2017). Homocysteine accumulates with age (Selhub, 1999; Tucker *et al*., 2005; Smith & Refsum, 2016) and is an independent risk factor for aging‐related diseases such as Alzheimer's disease (Clarke *et al*., 1998; Oulhaj *et al*., 2010; Beydoun *et al*., 2014; Smith & Refsum, 2016) and cardiovascular disease (Refsum *et al*., 1998). As homocysteine is a breakdown product of methionine, it is possible that homocysteine plays a role in the lifespan extension seen in mice in response to methionine‐restriction (Miller *et al*., 2005). It is likewise possible that its effects on mTOR signaling could be a contributing factor in this. In support of this hypothesis is the recent discovery that the LeuRS‐Flcn complex, which we described to be responsible for the homocysteine‐sensing of mTORC1, is associated with lifespan modulation (Nakamura *et al*., 2016).

Dietary changes do not need to be chronic to exert changes in longevity. A recent study showed that a transient high sugar diet in early adulthood can shorten lifespan in both *Drosophila* and *Caenorhabditis elegans*, via a transient inhibition of dFOXO/DAF‐16, causing short‐ and long‐term transcriptional changes (Dobson *et al*., 2017). This finding is in contrast with results that chronic excess sugar reduces lifespan in wild‐type and *dFOXO* mutants in equal measure (Al Saud *et al*., 2015), while *dFoxo* mutants still have a shortened lifespan versus wild‐types in general. It remains to be determined whether mTOR signaling is a mediator of the lifespan reduction caused by transient and chronic dietary sugar increases. It is possible that other transient nutritional changes can cause long‐term transcriptional or epigenetic changes affecting lifespan as well, and mTOR could be involved in mediating the changes, as has been shown for intermittent fasting (Honjoh *et al*., 2009).

## mTOR signaling in the control of food intake

A physiological nutrient‐sensing pathway through which mTOR could regulate metabolism and longevity is by acting as a behavior‐modulating ‘fuel sensor’. mTOR signaling has been shown to regulate food intake behavior through its activity in cells of the hypothalamus, a brain region important in the regulation of energy balance that integrates signals indicating the presence of a variety of nutrients, such as glucose, amino acids, lipids, and hormones such as leptin and insulin (Cota *et al*., 2006; Caron *et al*., 2016). Thus, mTOR activity is able to not only change intracellular metabolism, but also the behavior of the animal and global nutrient levels, raising further interesting questions about tissue‐specific mTOR activity in global homeostasis in metabolic disorders and aging, beyond the similarly organism‐wide effects mTOR signaling has in liver (Cornu *et al*., 2014; Lamming *et al*., 2014) and fat tissues (Polak *et al*., 2008; Cybulski *et al*., 2009; Kumar *et al*., 2010).

## Transcriptional regulation of cellular clearance

As discussed in detail in the previous section, mTORC1 is known to integrate many cellular signaling pathways to regulate protein and lipid synthesis, as well as clearance. Autophagy is an mTOR‐regulated intracellular, lysosome‐mediated clearance pathway for proteins and organelles. Autophagy function, including its initiation and ability to degrade sequestered cellular content, is referred to as autophagy flux. Autophagy flux is strictly dependent on nutritional status and lysosomal function, which directly affect mTORC1 activity and are regulated by the transcription factor EB (TFEB), a known mTORC1 substrate. TFEB regulates the activity of the coordinated lysosomal expression and regulation (CLEAR) gene network encoding for lysosomal and autophagosomal genes, thereby affecting the activity and number of degradative organelles (Sardiello *et al*., 2009; Settembre *et al*., 2012). Here, we review evidence for the contribution of autophagy to the lifespan modulating effects of dietary restriction and mTOR signaling.

Autophagy was first shown to be required for rapamycin‐induced, TOR‐mediated increase in longevity in yeast (Alvers *et al*., 2009). Rapamycin failed to increase lifespan when two genes required for autophagy were mutated, namely *atg1*, encoding for a protein serine/threonine kinase essential for autophagy, and *atg7*, essential for autophagosome formation, a necessary step in autophagic flux. The lifespan extension induced in *Caenorhabditis elegans* by reduced IIS was shown to be dependent on autophagy by silencing the gene *bec‐1* in a *daf‐2/INSR* loss‐of‐function background (Meléndez *et al*., 2003). *bec‐1* shares 31% homology with the human autophagy gene *beclin‐1*, which is required for functional autophagy. While the loss of *bec‐1* also decreased lifespan in wild‐type *Caenorhabditis elegans,* the effect was particularly dramatic in *daf‐2/INSR* mutants. Further evidence for the requirement of autophagy in IIS‐mediated lifespan extension was provided by a study using *daf‐2/INSR* mutants with two other autophagy‐related genes knocked down, namely *atg‐7* and *atg‐12* (Hars *et al*., 2007), both necessary for autophagy. Loss‐of‐function mutations in *bec‐1* and *atg‐7* lead also to the loss of lifespan extension in dietary restriction. Tóth *et al*. (2008) similarly examined autophagy gene involvement in *Drosophila* and *Caenorhabditis elegans* lifespan regulation, and in several longevity models for the latter, demonstrating that the need for functional autophagy in longevity strategies is not restricted to *Caenorhabditis elegans* or specific approaches. Still, autophagy alone is not sufficient for lifespan extension, but requires the simultaneous activity of DAF‐16/FOXO (Hansen *et al*., 2008), which engages programs that utilize the freed materials in the synthesis of protective, longevity enhancing proteins. The induction of autophagy by dietary restriction is dependent on PHA‐4/FOXA activity (Hansen *et al*., 2008), and PHA‐4/FOXA has been shown to initiate expression of genes involved in autophagy (Lapierre *et al*., 2011). As discussed, reduced IIS and mTOR inhibition can both activate DAF‐16/FOXO, in response to overlapping and distinct sets of cues, and mTOR signaling controls PHA‐4/FOXA activity. Thus, mTOR regulates at least three transcription factors involved in regulating autophagy and mediating the increase in lifespan provided by autophagy.

Several different models of increased lifespan in *Caenorhabditis elegans* require the process and genes of autophagy for their effects, including models for dietary restriction, reduced germline, insulin/IGF1 and TOR signaling, and mitochondrial respiration (Lapierre *et al*. 2011; Kapahi *et al*., 2016). As autophagy is controlled by mTOR both transcriptionally, via TFEB, and post‐transcriptionally, via ULK1, attenuated mTOR activity can enhance autophagy in low nutrient conditions. A central role in mediating the effects of autophagy on longevity has been described for TFEB. Analysis of factors governing *Caenorhabditis elegans* lifespan relating to autophagy has led to the discovery that the mTOR downstream gene *hlh‐30* is essential in six different models of increased lifespan (Lapierre *et al*., 2013), showcasing HLH‐30 as a key common transcription factor in *Caenorhabditis elegans* lifespan regulation across several distinct pathways. *hlh‐30* encodes for the *Caenorhabditis elegans* ortholog of the mammalian TFEB and has a conserved role as a regulator of autophagy also in *Caenorhabditis elegans* (Lapierre *et al*., 2013). A recently described autoregulatory feedback loop that induces TFEB expression during starvation and regulates lipid catabolism (Settembre *et al*., 2013) has also been shown to be conserved in *Caenorhabditis elegans*, demonstrating the long evolutionary roots of the transcriptional adaptive responses to starvation, and the central importance of autophagy in the regulation of cellular metabolism. Interestingly, TFEB localizes to the nucleus also in response to exercise causing calcium efflux from the sarcoplasmic reticulum and calcineurin‐mediated dephosphorylation of TFEB (Medina *et al*., 2015; Mansueto *et al*., 2017). TFEB controls metabolic flexibility in muscle during exercise, by regulating glucose uptake and glycogen content through transcriptional regulation of glucose transporters, glycolytic enzymes, and genes involved in mitochondrial biogenesis, fatty acid oxidation, and oxidative phosphorylation. This coordinated action optimizes mitochondrial substrate utilization, thus enhancing ATP production and exercise capacity. Physical activity, such as swimming, has been associated with the extension of healthspan in *Caenorhabditis elegans* (Chuang *et al*., 2016; Laranjeiro *et al*., 2017) and flies (Piazza *et al*., 2009)*,* as well as in mammals, including mice and humans (Cartee *et al*., 2016). Part of the beneficial effects of physical activity to lifespan could be mediated by TFEB. Examining lifespan changes caused by induced physical activity in *Caenorhabditis elegans* with a loss‐of‐function mutation in *hlh‐30* could determine whether this is the case. It remains to be determined whether autophagy induction by this transcription factor is a necessary component of lifespan increases caused by exercise.

## Control of proteostasis by TOR signaling

The other major cellular protein clearance pathway in addition to autophagy is the ubiquitin‐proteasome system. Recently, an mTOR‐linked pathway was discovered in *Caenorhabditis elegans* that signals from the lysosome to the nucleus and regulates lifespan via the proteasome. Folick *et al*. (2015) showed that increasing the expression of *lipl‐4*, a lysosomal acid lipase, increased lifespan, by activating nuclear hormone receptors NHR‐49 and NHR‐80 via nuclear localization of LBP‐8, a lysosomal lipid transporter. The authors also identified the probable lipid that is being transported, oleoylethanolamide, that is able to induce the expression of the nuclear hormone receptor target genes and bind directly to LBP‐8 and NHR‐10. Prior to this, O'Rourke & Ruvkun (2013) had shown a transcriptional link between nutrient levels and lysosomal lipolysis, also using *Caenorhabditis elegans* as a model. The presence of nutrients promotes the activity of the transcription factor MXL‐3, which suppresses the expression of genes driving lipolysis. Fasting represses *mxl‐3* transcription and promotes the nuclear localization of HLH‐30, inducing further *hlh‐30* expression and transcription of genes for lysosomal lipases and autophagy. This pathway provides another avenue by which TOR signaling can influence lifespan, as TOR inhibition increases LIPL‐4 expression and lipolysis (Lapierre *et al*., 2011). The TOR‐dependent increase in LIPL‐4 expression is also at least in part dependent on DAF‐16/FOXO, and IIS can contribute to increased LIPL‐4 expression via DAF‐2/INSR, again demonstrating convergence in lifespan modulation by nutrient‐sensing pathways.

Other ways TOR signaling can regulate proteostasis are by regulating protein translation through the kinase eIF4E, and by regulating the proteasome, a complex involved in the degradation of proteins in the ubiquitination pathway. Acute inhibition of TOR signaling increases protein degradation by the proteasome by enhancing ubiquitination of degradation targets (Zhao *et al*., 2015) and by a post‐transcriptional increase of levels of the proteasome and its chaperones (Rousseau & Bertolotti, 2016). Sustained inhibition of TOR signaling by genetic ablation of the TSC complexes, on the other hand, increases proteasome gene expression and protein turnover rates in a manner dependent on the transcription factor Nrf‐1 (Zhang *et al*., 2014). Additionally, TOR signaling could also affect transcriptional control of the proteasome via DAF‐16/FOXO, as this transcription factor has been shown to increase expression of *rpn‐6*, encoding a subunit of the 19S proteasome, in germline‐deficient *Caenorhabditis elegans* (Vilchez *et al*., 2012).

While it is established that TOR regulates cellular clearance via both autophagic and ubiquitin‐proteasome pathways, recently a third possible way through which cells can handle protein clearance was discovered in *Caenorhabditis elegans* (Melentijevic *et al*., 2017). Here, cells are able to spontaneously generate membrane‐bounded external organelles that are connected via a thin membranous capillary to the rest of the cell. These organelles, called exophers, appear to be used by cells to selectively extrude unwanted cargoes, including protein aggregates. This newly discovered clearance process is likely to promote longevity, as the established clearance pathways do, and to be a part of the mechanisms regulating proteostasis. Whether exopher formation and trafficking therein is controlled by nutrient‐ and stress‐sensing pathways and TOR signaling remains to be determined.

## Lipid metabolism: lipid synthesis, lipolysis, and lipophagy

Nutrient levels, insulin and growth factor signaling regulate lipogenesis, a process largely defined by the conversion of acetyl‐CoA to fatty acids. mTORC1 is a positive regulator of sterol regulatory element‐binding proteins (SREBPs), a family of transcription factors controlling fatty acid biosynthesis (Hua *et al*., 1993; Yokoyama *et al*., 1993; Wang *et al*., 1994; Eberlé *et al*., 2004), that is activated by PI3K/Akt signaling. Treatment with specific mTOR inhibitors, such as Torin, has been shown to lower expression levels of several genes involved in lipogenesis (Peng *et al*., 2002; Mauvoisin *et al*., 2007) by preventing the nuclear accumulation of SREBP in immortalized human retinal pigment epithelial cells (Porstmann *et al*., 2008). The SREBP/PI3K/Akt/TOR pathway is evolutionary conserved, as silencing the *Drosophila* ortholog of SREBP prevents the increase of cell growth by PI3K (Porstmann *et al*., 2008).

mTOR can regulate SREBP activity also by the phosphorylation of lipin 1, a phosphatidic acid phosphatase. It is important to emphasize that mTOR‐mediated regulation of SREBP activity is indirect and a consequence of changed nuclear morphology driven by the absence of lipin 1 from the nucleus. A loss‐of‐function mutation in the gene encoding lipin 1 results in fatty liver dystrophy in mice (Péterfy *et al*., 2001). Mice with this mutation are characterized by retarded growth, glucose intolerance, abnormal adipocyte differentiation, and hyperlipidemia. Huffman *et al*. (2002) demonstrated that lipin 1 is phosphorylated in response to insulin and that this phosphorylation is dependent on mTOR activity. Later, Peterson *et al*. (2011) showed that the mTOR‐mediated phosphorylation of lipin 1 regulates lipogenesis by inhibiting its nuclear localization. In the nucleus, enzymatic activity of lipin 1 changes the organization of the lamin A meshwork and nuclear morphology that is discussed to negatively affect SREBP presence in the nucleus. Indeed, the SREBP pathway is regulated by lamin A at different levels. Lamin A has been shown to physically interact with SREBP‐1 and SREBP‐2, and its expression can downregulate mRNA expression of the adipogenic SREBP target peroxisome proliferator‐activated receptor γ (PPARγ) (Lloyd *et al*., 2002; Boguslavsky *et al*., 2006).

Fatty acid synthesis, uptake, and esterification are controlled by the mTORC1‐regulated nuclear peroxisome proliferator‐activated receptors (PPARs) (Rosen & MacDougald, 2006). While the exact mechanism by which mTORC1 regulates nuclear PPARγ activity is still not fully understood, it has been shown that overexpression of the endogenous mTORC1 inhibitor Deptor increases adipogenesis and fat tissue buildup *in vivo* by inhibiting insulin signaling (Laplante *et al*., 2012). Active mTOR inhibits another PPAR, PPARα, and its co‐effector PGC1α, a nuclear receptor that controls the expression of genes required for fatty acid oxidation (Lefebvre *et al*., 2006). Inhibition of PPARα is promoted by the accumulation of nuclear receptor corepressor 1 (nCoR1), a negative regulator of several nuclear receptors. Kim *et al*. (2012) showed that S6 kinase 2 relays mTORC1 signals to nCoR1–PPARα, as deletion of S6 kinase 2 promotes ketone body formation in mice and high PPARα activity in cultured hepatocytes. Interestingly, it has been observed that mTORC1 activity is increased and PPARα activity reduced in aged mice (Okuda *et al*., 1987; Sastre *et al*., 1996; Sanguino *et al*., 2004; Sengupta *et al*., 2010) and inhibition of mTORC1 is sufficient to prevent aging‐related changes in PPARα activity (Sengupta *et al*., 2010). It is likely that the aging‐associated deregulation of mTORC1–PPARα signaling contributes to changes in systemic glucose and lipid homeostasis by impairing metabolic flexibility (Petersen *et al*., 2003).

As previously discussed, autophagy is upregulated in response to nutrient starvation and produces amino acids and glucose from proteins and glycogen, respectively. Autophagy plays a role also in the catabolism of lipids through a process known as lipophagy. When functional, lipid droplets are sequestered into autophagosomes and delivered to lysosomes for degradation (Singh *et al*., 2009). Lipophagy is part of basal lipid metabolism, as inhibition of this process leads to an accumulation of lipid droplets in cultured hepatocytes and mouse liver *in vivo*, and is especially important during dietary restriction. In *Caenorhabditis elegans*, it has been shown that animals lacking the lysosomal lipases LIPL‐1 and LIPL‐3 accumulate lipid droplets (O'Rourke & Ruvkun, 2013). Further, in *Caenorhabditis elegans*, both autophagy and lipolysis mediated by LIPL‐4 lipases are required for lifespan extension (O'Rourke & Ruvkun, 2013) thought to arise from the deviation of nutrients and energy resources from reproduction via reduced yolk lipoprotein production (Seah *et al*., 2016). Lipolysis is strongly dependent on autophagy and vice versa, as induction of autophagy by PHA‐4/FOXO requires LIPL‐4, and autophagy is required for maintained lipase activity in the germline‐deficient animals. Moreover, inhibition of mTOR, upstream of PHA‐4/FOXO‐ and HLH‐30/TFEB‐dependent autophagy induction, also causes upregulation of LIPL‐4 expression. TFEB target genes include the sole mammalian lysosomal lipase LAL (Palmieri *et al*., 2011), suggesting further that the transcriptional regulation of lysosomal lipolysis is at least in part achieved by transcription factors controlling autophagy, and that the processes are linked to drive lipophagy.

Recently, mTOR signaling was implicated by Han *et al*. (2017) as a component in lifespan affecting, histone methylation‐controlled changes in mono‐unsaturated fatty acid (MUFA) metabolism in *Caenorhabditis elegans*. H3K4me3 methyltransferase deficiency in the germline leads to an increase in longevity that is dependent on the intestinal accumulation of MUFAs. This deficiency also downregulates the mTOR substrate *rsks‐1*/S6K. The authors demonstrated that deficiencies in *rsks‐1*/S6K or its upstream regulators *let‐363*/mTOR or *daf15*/Raptor are sufficient to induce similar changes, resulting in intestinal MUFA accumulation and increased lifespan, which was not additive with the methyltransferase deficiency. While the authors implicate SBP‐1/SREBP as a transcription factor responsible for the MUFA accumulation for the methyltransferase deficiency, it is not clear whether this is the case also for the effects seen in *rsks‐1*/S6K1‐deficient worms, as *rsks‐1*/S6K1 deficiency did not lead to the nuclear accumulation of SBP‐1/SREBP. Thus, how deficiencies in TOR‐signaling components result in MUFA accumulation remains unknown. Put into a broader context of mTOR‐mediated lifespan regulation, this study raises the interesting question whether part of the lifespan increase provided by mTOR inhibition is due to the resultant changes in fatty acid metabolism that accumulate endogenous MUFAs, and whether this is a pathway that is conserved in mammals.

## Sestrins and TSC1/2 – Upstream regulators of mTORC1 and associated transcription factors

As several strategies of genetic, pharmacological, and nutritional lifespan extension require TOR signaling, it is interesting to assess the role and specificity of upstream regulators of the mTOR complexes that are affected by said approaches. Sestrins are a conserved family of cellular metabolism regulating proteins that enhance AMPK signaling and inhibit TOR signaling in response to amino acid starvation, and whose expression is induced by stress. Sestrins were first shown to have a role in aging processes in *Drosophila* (Lee *et al*., 2010b), in which the loss of its only sestrin ortholog, *dSesn*, leads to age‐associated pathologies in muscle tissue, heart function, triglyceride homeostasis, and mitochondria. In this study, *dSesn* was described as a feedback inhibitor of TOR signaling, as chronic TOR activation increased the levels of *dSesn* expression in a reactive oxygen species (ROS), c‐Jun amino‐terminal kinase, and FOXO‐dependent manner. Other studies have provided evidence for the conserved roles of sestrins in regulating processes related to aging. Yang *et al*. (2013b) showed that *sesn‐1/Sesn*, the only sestrin ortholog in *Caenorhabditis elegans*, positively regulates lifespan in the nematode. Overexpression of *Sesn* increased the lifespan of the animals, and loss‐of‐function mutants had elevated levels of ROS as well as reduced function of muscle tissues, similar to the results in *Drosophila*.

Mouse mutant studies have shown roles for Sestrins in mammalian cellular homeostasis. Sestrins 1 and 2 were shown to protect the liver from oxidation by activating Nrf2, a transcription factor for antioxidants, via reducing levels of its suppressor Keap1 by means of p62‐dependent autophagy (Bae *et al*., 2013). Here Sesn2 was shown to be upregulated by fasting, and to interact with p62, an autophagy substrate, and Keap1, the target of degradation. Importantly, sestrins can also suppress the activity of TOR signaling in mice. In a study of obesity‐related metabolism (Lee *et al*., 2012), loss of Sestrin 2 caused further TOR activation in the liver, glucose intolerance and insulin resistance in obese mice, and loss of sestrins 2 and 3 resulted in insulin resistance and TOR activation in mice on a normal diet.

Of special interest is the TSC1/2 mTOR inhibitor complex, as it incorporates signals from several pathways to regulate mTORC1 activity, including from IIS via Akt, stress signaling via ERK, energy sensing via AMPK, hypoxia via Redd1, and cytokines via IKKB and NFkB (Huang & Manning, 2008). Depletion of TSC1 and TSC2 has been shown to decrease lifespan, and overexpression of TSC2 in fat tissue to increase lifespan in *Drosophila* (Kapahi *et al*., 2004). A recent study demonstrated that overexpression of TSC1 in mice extends their lifespan and healthspan (Zhang *et al*., 2017). Notably, mTORC1 signaling was not reduced in all tissues. The brain was resistant to the reduction, possibly due to its elevated basal levels of TSC1 expression. Likewise of note is that the healthspan effect was seen only in female mice, raising further questions about the gender‐specificity of mTORC1 signaling effects on longevity, which has been shown before. The mechanisms of this specificity remain a subject for future studies. mTORC2 activity, on the other hand, was increased by TSC1 overexpression, demonstrating how the two mTOR complexes can have opposite activity levels. It would be interesting to see whether the increased mTORC2 activity is necessary for the lifespan extension provided by TSC1 overexpression. While both sestrins and TSC1/2 have been associated with mTOR‐mediated longevity, a further interesting and unanswered question is whether their effects are fully dependent on mTOR, or if they have mTOR‐independent functions in regulating lifespan and healthspan. Evidence exists for at least TSC1 of such mTOR‐independent cellular effects (Zhu *et al*., 2014).

## Induced pluripotent stem cells, *in vivo* reprogramming and metabolism

The reprogramming of somatic cells by overexpression of four specific factors Oct4, Sox2, Klf4, and c‐Myc (OSKM) into patient‐specific stem cells (Takahashi & Yamanaka, 2006), the so‐called induced pluripotent stem cells (iPSCs), and their differentiation into disease‐relevant cell types, offer previously unanticipated opportunities to model age‐related human disease in a culture dish. The use of this model system presents new challenges and clear advantages when compared to systems with nearly identical genetic backgrounds. One of these new possibilities is the ability to address the impact of diverse genetic backgrounds on cellular metabolism, including receptor‐mediated and lysosomal mTOR signaling. To achieve this, a common approach is the generation of isogenic iPSC line pairs, with and without mutating the selected gene/s using genome editing techniques or simply by increasing the number of control and patient‐specific cell lines. However, testing for diverse genetic backgrounds can be facilitated to determine the genetic penetrance of a given mutation, a too rarely determined phenomenon in molecular biology. Besides the testing for a variety of genetic backgrounds, iPSC‐derived human cells allow a high degree of experimental freedom, and avoidance of the overexpression of human, aggregation‐prone proteins, which is commonly done in ‘humanized’ mouse models.

Age is the main risk factor in the development of various human diseases such as cancer, neurodegeneration, or cardiovascular disease. Therefore, modeling aging with disease‐relevant cell types is an important and constantly evolving approach to understanding the mechanisms of age‐related disease. Generation of an iPSC line carrying a distinct mutation of the nuclear *lamin A/C* gene derived from patients with atypical Werner syndrome and Hutchinson Gilford progeria syndrome (HGPS) was among the first approaches to mimicking premature aging phenotypes in iPSC‐derived cell types (Ho *et al*., 2011). Follow‐up studies involved overexpression of the truncated form of lamin A, also known as progerin, to induce aging‐related phenotypes, such as late‐onset Parkinson's disease involving dopamine deficiency and neuromelanin accumulation, an aging‐related feature usually not observed in iPSC‐derived models (Miller, 2013). It has been generally accepted until recently that iPSCs are highly similar to embryonic stem cells and can be propagated in culture infinitely. However, it has been shown that iPSCs indeed age in culture and have reduced expression of mitochondrial genes and their metabolic status over time (Masotti *et al*., 2014). On the other hand, studies from the Gage laboratory show that direct reprogramming of somatic cells into induced neurons preserves age‐specific gene signatures, while their conversion to iPSCs diminishes these changes (Mertens *et al*., 2015). As a novel age‐associated phenotype, this study identified a loss of nucleocytoplasmic compartmentalization driven by low levels of the nuclear transport receptor RanBP17; its role in maintaining homeostatic FOXO and TFEB signaling, and therefore in autophagy‐related gene expression, remains to be determined. In contrast to the findings by the Gage group, a recent study showed that the age‐related epigenetic signature depends on donor's age and is retained in iPSCs and can be reduced through passaging (Sardo *et al*., 2016). In this study, iPSC lines from 16 donors aged 21‐100 were generated, and epigenetic and whole genome sequencing were conducted to identify somatic mutations in individual donor cells, a method made possible by clonal expansion during the reprogramming process. These elegantly designed analyses demonstrate that exomic mutations in iPSCs increase linearly with age, with elderly donors older than 90 years harboring fewer mutations than predicted, and all analyzed lines carrying at least one pathogenic mutation. This study shows that age increases the risk of genetic abnormalities in iPSCs, which can be used as a clinical measure in the development of future reprogramming techniques. Modeling late‐onset neurodegenerative disorders, such as Parkinson's, Alzheimer's, or Gaucher diseases, has provided initial insight into the contributions of the regulation of metabolic signaling to pathogenesis. In all of these three diseases, TFEB and TFE3 signaling are deregulated, and activation of the pathway prevents (or, protects from) mitochondrial dysfunction, buildup of abnormal proteins, and neurodegeneration (Awad *et al*., 2015; Siddiqui *et al*., 2015; Khayati *et al*., 2017). While iPSC‐derived human cell system is becoming an essential tool in studying age‐related human diseases, recent studies show that transient overexpression of OSKM reprogramming factors *in vivo* mitigates the hallmarks of aging (Ocampo *et al*., 2016). Furthermore, in the same study, the OSKM‐induced *in vivo* reprogramming prolongs the lifespan of a progeria mouse model and improved muscle regeneration after injury and recovery from metabolic disease in older wild‐type mice (Ocampo *et al*., 2016). These exciting findings described by Ocampo *et al*. underline the importance of epigenetic changes driving the aging process and further link these to metabolic pathways discussed here. The association between epigenetic state, cell fate, and cellular metabolism has been discussed elsewhere (Wu *et al*., 2016), but how chromatin modifications and metabolic states are integrated remains largely unknown. It is possible that epigenetic changes can regulate activity of mTOR or associated proteins, such as S6K1, either directly through histone methyltransferase complexes (Han *et al*., 2017) or transcription factors such as TFEB or FOXO.

## Conclusions and future directions

mTOR is an integrative hub at the center of several conserved, nutrient‐sensing pathways that affect longevity by several means, including by transcriptional regulation of processes such as autophagy, the focus of this review. The mechanism through which mTOR accelerates cellular and organismal aging is still unclear, but causative elements discussed include increased oxidative and proteotoxic stress associated with mTOR‐mediated mRNA translation (Selman *et al*., 2009) and inhibition of autophagy (Rubinsztein *et al*., 2011) resulting in the accumulation of defective organelles, including mitochondria. It is important to emphasize the complexity of the pathway: mTOR regulates metabolic transcription factors and can be regulated by the same transcription factors, such as TFEB and FOXO, and mTOR is able to regulate nuclear morphology and induce epigenetic changes by which it is affected (Peterson *et al*., 2011).

Several components of the mTOR pathway have still not been investigated in the context of aging and longevity, such as CASTOR and the recently discovered KICSTOR, a protein complex composed of KPTN, ITFG2, C12orf66, and SZT2, that is required to inhibit mTORC1 during amino acid or glucose deprivation. It is possible that differential expression or activity of TOR‐regulating proteins can be part of the age‐associated changes in the base level of mTOR signaling, associated also with a decline in protein turnover and autophagy, and increase in protein aggregation (David *et al*., 2010; Laplante & Sabatini, 2012; Lapierre *et al*., 2015). Another possible way the regulation of these longevity‐driving processes could deteriorate over time is the loss of nucleocytoplasmic compartmentalization, as seen in progeria (Mertens *et al*., 2015; Soria‐Valles & López‐Otín, 2016), and also in healthy aged individuals, whose cells show evidence of increased nuclear membrane blebbing and progerin buildup (Mertens *et al*., 2015). In addition to the recorded effects of this loss on DNA damage and promotion of cellular senescence, further aggravated with simultaneously increased mTOR signaling, this could possibly disable the highly controlled localization of transcription factors, including those regulating processes related to aging, feeding into a vicious cycle of perturbed metabolism and homeostasis.

Rapamycin has recently been shown to alleviate some aging phenotypes while exacerbating others (Wilkinson *et al*., 2012; Neff *et al*., 2013). These results could be due at least in part to attenuated mTORC2 activity, the loss of which has been shown to reduce longevity in *Caenorhabditis elegans* (Mizunuma *et al*., 2014) and in liver‐specific mTORC2 knockout mice (Lamming *et al*., 2014), while inhibition of mTORC1 is largely viewed as advantageous. Development of new drugs targeting the amino acid sensing pathway may increase selectivity to mTORC1 and enable assessments of longevity changes upon pharmacological complex‐specific mTOR inhibition. One promising group of potential new drugs are amino acid metabolites. These naturally occurring compounds bind to cellular amino acid sensors that regulate mTORC1 activity. A recent example has been published by our group for leucinol, a leucine metabolite that binds and inhibits LeuRS and Flcn (Khayati *et al*., 2017). In addition to the LeuRS/Flcn complex, other cellular amino acid sensors should be targeted by amino acid metabolites as well. Two known sensors are Sestrin 2 that senses the availability of leucine, methionine, isoleucine and valine (Wolfson *et al*., 2015), and CASTOR, a cellular arginine sensor (Chantranupong *et al*., 2016); the sensitivity of mTORC1 toward metabolites of these amino acids and their possible lifespan effects remain to be determined in future studies. The longevity pathways described in this review were originally discovered in studies in which the signaling has been perturbed in the whole organism, missing information on whether regional differences and regional changes in signaling over time play a role in aging, with some organs being more important than others. More recently, researchers have started to address these metabolism and lifespan regulating pathways in different tissues. The most common approach to studying aging has been to use species with limited lifespans, such as *Caenorhabditis elegans*,* Drosophila* and mice, and measuring longevity upon different treatments and genetic backgrounds. However, as recently discussed among scientists and medical professionals, a better measurement could be healthspan, a quantitative assessment of cell and tissue homeostasis and function. An extended healthspan, and not merely extension of lifespan, is indeed the aim of medical practice. Techniques to assess physical performance during aging are currently being developed. Development of these new techniques and pharmacological agents targeting specific inputs, components, and outputs of the longevity‐modulating pathways, as well as recent advances made in human iPSC technology, will foster the search for mechanisms of and interventions against aging‐related diseases and the aging process.

## Funding

This work was supported by the Presidential Graduate Award to HPA and the American Federation for Aging Research #RAG13447 to RD.

## Conflict of interest

None declared.
